# miR-135b-5p enhances doxorubicin-sensitivity of breast cancer cells through targeting anterior gradient 2

**DOI:** 10.1186/s13046-019-1024-3

**Published:** 2019-01-21

**Authors:** Ying Zhang, Fan Xia, Fan Zhang, Yingying Cui, Qingling Wang, Hui Liu, Yongping Wu

**Affiliations:** 1 Department of Pathology, Laboratory of Clinical and Experimental Pathology, Xuzhou Medical University, No. 209, Tongshan Rd, Xuzhou, 221004 China; 2 Key Laboratory of Bone Marrow Stem Cell of Jiangsu Province, Xuzhou Medical University, No. 84, Huaihaixi Rd, Xuzhou, 221002 China; 3 Department of Pathology, The Affiliated Hospital of Xuzhou Medical University, No. 99, Huaihaixi Rd, Xuzhou, 221002 China

**Keywords:** Breast cancer, Anterior gradient 2, microRNA, Doxorubicin

## Abstract

**Background:**

The pro-oncogenic anterior gradient 2 (AGR2) is involved in tumor growth and drug resistance of breast cancer. Mechanisms that regulate expression of AGR2 still need to be elucidated.

**Methods:**

In this study, expression levels of AGR2 and miR-135b-5p were analyzed in different breast cancer cell lines as well as in clinical breast cancer tissues. The in vitro and in vivo functional effect of AGR2 and miR-135b-5p were also investigated. A luciferase reporter assay was applied to confirm the interaction between miR-135b-5p and *AGR2* mRNA.

**Results:**

We identified AGR2 as a target of miR-135b-5p. Expression of AGR2 was up-regulated in doxorubicin-resistant breast cancer cells. AGR2 mediated doxorubicin-sensitivity of breast cancer cells both in vitro and in vivo. miR-135b-5p negatively regulated AGR2-expression of breast cancer cells increasing doxorubicin-sensitivity. However, miR-135b-5p was down-regulated in doxorubicin-resistant breast cancer cells as well as during treatment with doxorubicin, which might be a probable reason for over-expression of AGR2. Up-regulation of miR-135b-5p increased doxorubicin-sensitivity of breast cancer cells in vivo. In addition, levels of AGR2 negatively correlated with levels of miR-135b-5p in clinical breast cancer tissue samples.

**Conclusion:**

Our results highlight the potential of miR-135b-5p as a target for treating AGR2-expressing breast cancer with doxorubicin-resistance.

**Electronic supplementary material:**

The online version of this article (10.1186/s13046-019-1024-3) contains supplementary material, which is available to authorized users.

## Background

Breast cancer is one of the most common malignant tumors worldwide [1, 2]. Although surgical treatments are curative for some early stage cases, adjuvant systemic therapies are important for improving survival of patients especially those who with advanced stage diseases [3]. Doxorubicin, a topoisomerase II inhibitor, is a backbone drug in most chemotherapeutic regimens [4]. However, intrinsic or acquired drug resistance to doxorubicin limited the efficacy of doxorubicin-based treatments [5, 6]. Mechanisms mediating drug resistance to doxorubicin still need to be elucidated.

The human anterior gradient 2 (AGR2), a member of protein disulphide isomerases (PDIs) family, regulates protein folding in endoplasmic reticulum and normal mammary gland development [7, 8]. Over-expression of AGR2 is involved in pathogenesis of breast cancer including growth, drug resistance and metastasis of tumors, which is associated with poor prognosis [9]. Intrinsic or acquired over-expression of AGR2 was shown to mediate resistance to hormone therapies in estrogen receptor (ER)-positive breast cancers [10]. AGR2 was also implied to mediate doxorubicin resistance in breast cancer cells [11]. These findings suggest that AGR2 is an important mediator of drug resistance in breast cancer. *AGR2* was shown to be a target of ER, which regulates expression of AGR2 in both normal mammary gland and breast cancer [12, 13]. However, over-expression of AGR2 is not restricted to ER-positive breast cancer. High AGR2 expression could be observed in ER-negative breast cancers, while some ER-positive cases showed low levels of AGR2 suggesting that mechanisms other than ER might control expression of AGR2 in breast cancer [10].

MicroRNAs (miRNAs) are single strand non-coding RNAs which regulate expression of genes at post-transcriptional level through binding 3′-untranslated region (3′-UTR) of mRNA. Some reports had shown that decreased levels of miRNAs led to over-expression of specific oncogenes promoting pathogenesis of cancers [14, 15]. Aberrant levels of miRNAs were also recognized as predictive factors of drug resistance in breast cancer [16]. Based on the important roles of AGR2 and miRNAs in breast cancer, we interrogated how miRNAs regulate expression of AGR2 in breast cancer cells. In this study, we found AGR2 was up-regulated in doxorubicin-resistant breast cancer cells. miR-135b-5p negatively regulates expression of *AGR2* which increased sensitivity to doxorubicin in breast cancer cells both in vitro and in vivo. Our finding is indicative for an important role of miR-135b-5p/AGR2 pathway in regulating doxorubicin-sensitivity of breast cancer cells.

## Methods

### Clinical breast cancer specimens

Twenty-eight breast cancer samples were collected at the Affiliated Hospital of Xuzhou Medical University between October 2017 and April 2018. Subject and disease related variables are shown in Table 1. All the patients have not being treated before resection.

**Table 1:** Clinical and pathological information of patients

Number	ID	Age (year)	Tsize (mm)	Lymph node metastasis	Grade	AJCC anatomic stage	ER	PR	HER2	Subtype
1	1,595,476	35	30	0	3	2A	N	N	N	Triple negative
2	1,597,702	44	25	0	3	2A	N	N	N	Triple negative
3	1,613,380	39	25	0	3	2A	N	N	N	Triple negative
4	1,561,558	47	30	0	2	2A	N	N	N	Triple negative
5	1,593,213	71	30	0	1	2A	N	N	N	Triple negative
6	1,558,369	67	45	2	3	2B	N	N	P	Her2 overexpressing
7	1,562,676	46	22	0	3	2A	N	N	P	Her2 overexpressing
8	1,568,640	72	25	3	3	2B	N	N	P	Her2 overexpressing
9	1,538,312	73	15	0	2	1A	P	P	N	Luminal A
10	1,538,856	59	40	3	3	2B	P	P	N	Luminal A
11	1,540,806	54	30	1	2	2B	P	P	N	Luminal A
12	1,557,688	61	15	0	2	1A	P	P	N	Luminal A
13	1,563,656	43	30	1	3	2B	P	P	N	Luminal A
14	1,594,881	44	20	0	2	1A	P	P	N	Luminal A
15	1,558,066	49	15	0	2	1A	P	P	N	Luminal A
16	1,547,013	62	15	0	1	1A	P	P	N	Luminal A
17	1,551,621	49	20	0	2	2A	P	P	N	Luminal A
18	1,554,600	74	20	2	3	2B	P	P	N	Luminal A
19	1,540,752	48	20	1	2	2B	P	P	P	Luminal B
20	1,545,637	44	50	13	3	3C	P	P	P	Luminal B
21	1,589,813	35	18	1	2	2A	P	P	N	Luminal A
22	1,595,773	64	27	0	1	2A	P	P	N	Luminal A
23	1,595,546	47	30	13	3	4	P	P	P	Luminal B
24	1,596,351	45	63	0	3	2B	P	P	P	Luminal B
25	1,608,794	39	25	0	3	2A	P	P	N	Luminal A
26	1,609,339	52	30	12	3	3C	P	P	N	Luminal A
27	1,612,095	67	20	3	2	2B	P	P	N	Luminal A
28	1,595,081	39	30	22	3	3C	P	P	N	Luminal A

*AJCC* American Joint Committee on Cancer, *ER* estrogen receptor, *HER2* human epidermal growth factor receptor 2, *N* negative, *P* positive, *PR* progesterone receptor, *Tsize* tumor size

### Mice

BALB/c Nude mice were purchased from Vital River (Charles River, Beijing, China). Mice were bred in a special pathogen free room.

### Cell culture

MCF-7 cells (ATCC HTB-22) were cultured in DMEM medium (Thermo Fisher Scientific, Waltham, MA, USA) supplied with 10% FBS (Biowest, Nuaillé, France), penicillin and streptomycin. MDA-MB-231 (ATCC HTB-26) cells were cultured in Leibovitz’s L-15 medium (Thermo Fisher Scientific) supplied with 10% FBS, penicillin and streptomycin. MDA-MB-231 cells were maintained without CO_2_ equilibration.

Doxorubicin-resistant MCF-7 cells (MCF-7/DOXR) were selected as previously described [17]. MCF-7 cells were sequentially exposed to increasing doses of doxorubicin (0.1, 0.5, 1.0, 2.0 and 5.0 μM). Cells were initially cultured in DMEM medium with 0.1 μM doxorubicin for 1 d, followed by culture with doxorubicin free DMEM medium for 4 d. Selection with the same concentration of doxorubicin was repeated twice before moving to selection with the next dose.

### Reagents

Doxorubicin, paclitaxel, docetaxel and 4-hydroperoxy cyclophosphamide were purchased from ApexBio (Houston, TX, USA). Puromycin was purchased from Sigma-Aldrich (Shanghai, China).

### Quantitative polymerase chain reaction (qPCR)

Relative expression level of *AGR2* mRNA was detected using qPCR as described previously [18]. Total RNA was isolated using TRIzol reagent (Invitrogen, Thermo Fisher Scientific). cDNA was synthesized with a PrimeScript cDNA Synthesis Kit (Takara Bio Inc., Shiga, Japan) followed analysis with a LightCycler 480 SYBR Green I Master qRT-PCR kit (Roche, Mannheim, Germany). *ACTB* was used as a normalization gene. The following primers were synthesized from Invitrogen (Thermo Fisher Scientific, Shanghai, China): *AGR2* (GTGTAGGAGAGGGCCACAAG and CGACTCACACAAGGCAGGT) and *ACTB* (GTTGTCGACGACGAGCG and GCACAGAGCCTCGCCTT).

For detecting expression levels of mature miRNAs, cDNA was synthesized from total RNA using a miScript II RT Kit (QIAGEN, Shanghai, China). qPCR was performed using a miScript SYBR Green PCR Kit (QIAGEN) with U6 as a normalization gene. The following primers were used: miR-342-3p (Forward: TCTCACACAGAAATCGCACCCGT), miR-217 (Forward: TACTGCATCAGGAACTGATTGGA), miR-135b-5p (Forward: TATGGCTTTTCATTCCTATGTGA), miR-194-5p (Forward: TGTAACAGCAACTCCATGTGGA), miR-543 (Forward: AAACATTCGCGGTGCACTTCTT), miR-24-3p (Forward: TGGCTCAGTTCAGCAGGAACAG), miR-377-3p (Forward: ATCACACAAAGGCAACTTTTGT), miR-3158-3p (Forward: AAGGGCTTCCTCTCTGCAGGAC), miR-216b-3p (Forward: ACACACTTACCCGTAGAGATTCTA), miR-124-5p (Forward: CGTGTTCACAGCGGACCTTGAT), miR-1267 (Forward: CCTGTTGAAGTGTAATCCCCA), miR-624-3p (Forward: CACAAGGTATTGGTATTACCT) and U6 (CTCGCTTCGGCAGCACA and AACGCTTCACGAATTTGCGT). The results were analyzed by using the method of comparison on –ΔΔct values. qPCR was performed on an LC480 cycler (Roche).

### Western blot analysis

Proteins were extracted from cells or tissues using Cell Lysis Buffer (Cell Signaling Technology, Danvers, MA, USA). Western blot analyses were performed with the following antibodies: Cleaved Caspase-3 (Asp175), Mcl-1 (D2W9E), Bcl-2 (D17C4), Bcl-xL (54H6), Bim (C34C5), Bak (D4E4), Cyclin D1 (92G2), Cyclin E1 (D7T3U), CDK2 (78B2), CDK4 (D9G3E), CDK6 (D4S8S), AGR2 (D9V2F) and GAPDH (D16H11), all of which were purchased from Cell Signaling Technology.

### Cell viability assay

Cell viability was detected using a Cell Counting Kit-8 (CCK-8) from DOJINDO (CK04, Tokyo, Japan). Briefly, cells were incubated with CCK-8 reagent for 1 h followed by reading optical density at 450 nm.

### Short hairpin RNA (shRNA)-mediated knockdown of AGR2

Three pairs of human *AGR2*-specific shRNAs (*AGR2*-shRNA) were synthesized according to the human *AGR2* gene sequence (NM_006408). An unrelated negative shRNA was used as control. All shRNAs were cloned to a pLVX-shRNA2-Puro lentiviral vector (Genecreate, Wuhan, China). Lentiviral shRNA particles were obtained by transfecting 293FT cells with the constructed lentiviral vector packed in a ViraPower HiPerform Lentiviral Expression System (Invitrogen, Thermo Fisher Scientific). MCF-7 cell line, stably expressing *AGR2*-shRNA, was established by transducing cells with lentiviral shRNA particles, followed by selection with puromycin. Our preliminary experiment identified one of the *AGR2*-shRNA as the most efficient one in silencing AGR2-expression.

### Over-expression of AGR2

CDS fragment (528 bp) of human *AGR2* (NM_006408) was synthesized and cloned to pCDH-CMV-MCS-EF1-GFP-Puro lentiviral vector (Genecreate). Lentiviral AGR2 particles were obtained as described above. MCF-7 and MDA-MB-231 cell lines, over-expressing AGR2, were established as described above. A blank vector was used as control.

### Xenograft experiments

BALB/c Nude mice were anesthetized and inoculated subcutaneously with 5 × 10^6^ gene modified MCF-7 cells. Mice were injected intraperitoneally (i.p.) with PBS or doxorubicin (5 mg/kg) once on day 12, 15 and 18 after cell inoculation. Tumor size was measured continuously. Tumor volume was calculated with the formula: Tumor volume (mm^3^) = length (mm) × width (mm) × width (mm) × 0.5. Tumors were collected on day 32 for histological analyses.

### H&E staining and immunohistochemistry

Tissue slides were prepared from tumors collected from xenograft experiments. H&E staining was performed as previously described [19].

Immunohistochemistry was performed to detect expression of Ki-67 and cleaved caspase-3 in tumor tissues. Anti-Ki-67 and peroxidase-conjugated goat anti-rabbit IgG were from ZSGB-BIO (Beijing, China). Anti-cleaved caspase-3 (Asp175) was from Cell Signaling Technology. DAB substrate was used for visualization (ZSGB-BIO).

### TUNEL assay

TUNEL assay was performed to detect apoptotic cells in tissue slides using a TUNEL Kit per manufacturer’s instructions (KGA702, KeyGEN BioTECH, Beijing, China).

### β-Galactosidase staining

Senescence of cells was analyzed using a β-Galactosidase Staining Kit per manufacturer’s instructions (KGPAG001, KeyGEN BioTECH).

### Prediction of miRNA target

Several candidate miRNAs were predicted and selected from literature [20] and miRNA databases (TargetScan and miRBase). All these candidate miRNAs had a potential targeting 3′-UTR of *AGR2* mRNA (Additional file 1 Table S1).

### Luciferase reporter assay

3′-UTR (1097 bp) of *AGR2* mRNA was synthesized and cloned to pmirGLO Dual-Luciferase miRNA Target Expression Vector (Promega, Madison, WI, USA). A mutant *AGR2* 3′-UTR (1090 bp) luciferase vector, lacking the binding site AAGCCAT of miR-135b-5p, was also constructed. Luciferase vector and miRNA mimics (Genecreate) were co-transfected into MCF-7 cells using Attractene Transfection Reagent (QIAGEN). Twenty-four hours after transfection, firefly and renilla luciferase activities of cell lysate were analyzed by using a Dual-Luciferase Reporter Assay kit (Promega). Firefly luciferase is a reporter, and renilla luciferase is a normalizer.

### Lentivirus mediated over-expression of miR-135b

Lentiviral particles, expressing a control miRNA or hsa-miR-135b double strand precursors, were obtained from Genechem (Shanghai, China). MCF-7 cell lines, stably expressing control miRNA or hsa-miR-135b, were established by transducing cells with lentiviral particles, followed by selection with puromycin.

### RNA interference

*ESR1*-targeting small interfering RNAs (siRNA) (*ESR1* siRNA-1: CCGGCAUUCUACAGGCCAA, *ESR1* siRNA-2: GGAGAAUGUUGAAACACAA, *ESR1* siRNA-3: GCUAGAGAUCCUGAUGAUU), synthesized from Genecreate (Wuhan, China), were transfected into MCF-7 cells using HiPerFect Transfection Reagent (QIAGEN). An irrelevant siRNA (Genecreate) was used as negative control.

### Statistics

Data are presented as mean ± standard deviation (SD). Comparison of means was performed with unpaired Student *t* test or one-way ANOVA test followed by Bonferroni post-tests. Correlations were analyzed using *Pearson* correlation test. Value of *p* < 0.05 was considered statistically significant.

## Results

### Knockdown of AGR2 increased doxorubicin-sensitivity of breast cancer cells in association with enhanced apoptosis

**Fig. 1:**
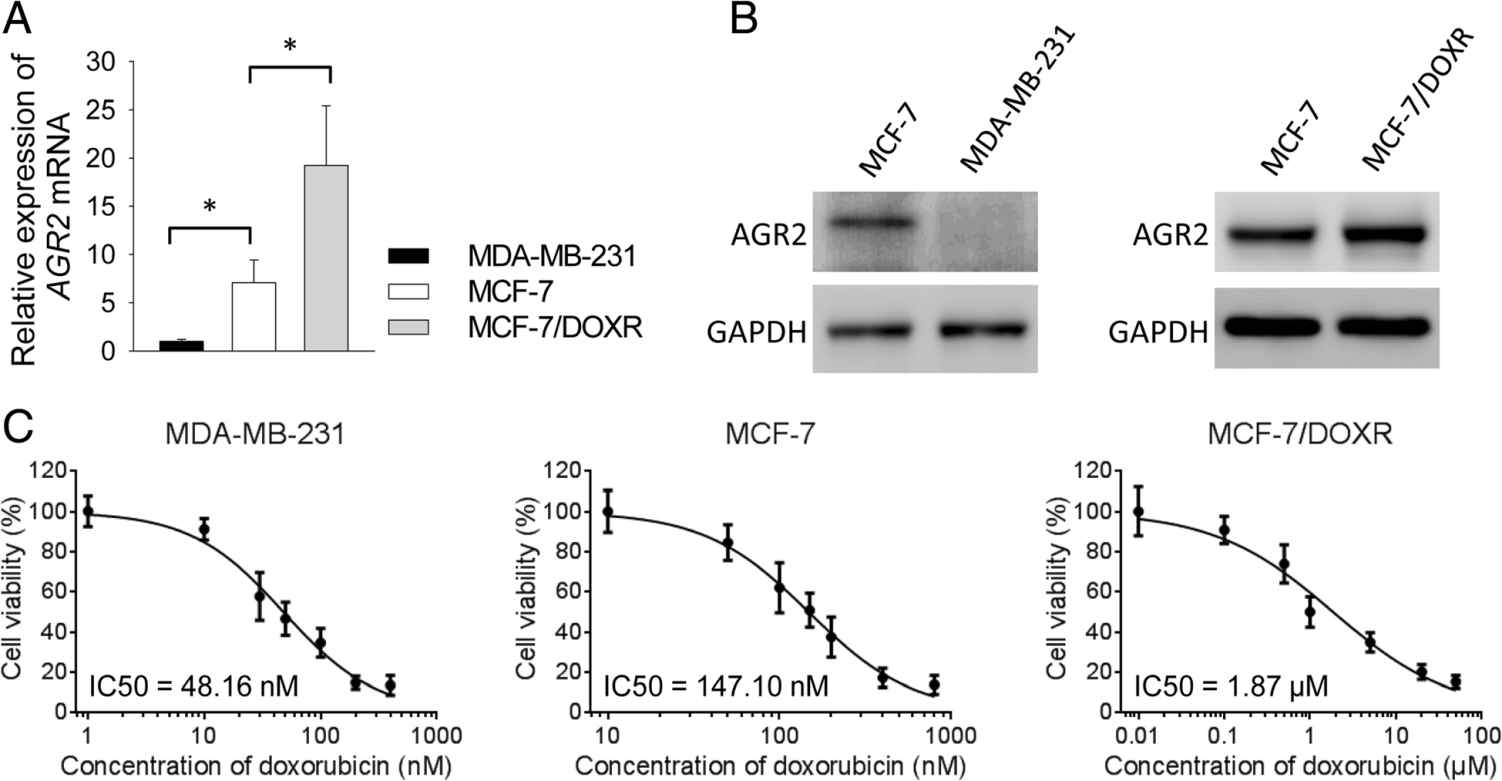
AGR2 correlated with doxorubicin-resistance in breast cancer cells. **a**-**b** Total RNA and protein were extracted from MDA-MB-231, MCF-7 and MCF-7/DOXR cells. **a** qPCR and **b** western blot were applied to analyze mRNA and protein levels of AGR2. **c** Cells were treated with gradient doses of doxorubicin for 48 h. MDA-MB-231: 1, 10, 30, 50, 100, 200, 400 nM; MCF-7: 10, 50, 100, 150, 200, 400, 800 nM; MCF-7/DOXR: 0.01, 0.1, 0.5, 1.0, 5.0, 20, 50 μM. Cell viability was measured by CCK-8 assay (*n* = 6). Viability of untreated cells was set as 100%. Experiment was repeated twice. Expression levels of mRNA represent fold changes. Data are shown as mean ± SD, compared using one-way ANOVA test. *, *p* < 0.05; MCF-7/DOXR, doxorubicin-resistant MCF-7 cells

To assess the correlation between expression of AGR2 and doxorubicin-sensitivity in breast cancer cells, we analyzed expression of AGR2 mRNA in MDA-MB-231 and MCF-7 cells as well as doxorubicin-resistant MCF-7/DOXR cells. MCF-7 cells expressed higher levels of AGR2 than MDA-MB-231 cells at both mRNA and protein levels. Expression of AGR2 protein was not observed in MDA-MB-231 cells, although we could detect low level of *AGR2* mRNA in this cell line (Fig. 1a and b). Interestingly, expression of AGR2 was up-regulated in MCF-7/DOXR cells as compared with MCF-7 cells (Fig. 1a and b). IC50 of doxorubicin in MDA-MB-231, MCF-7 and MCF-7/DOXR cells was 48.16 nM, 147.10 nM and 1.87 μM, respectively (Fig. 1c).

**Fig. 2:**
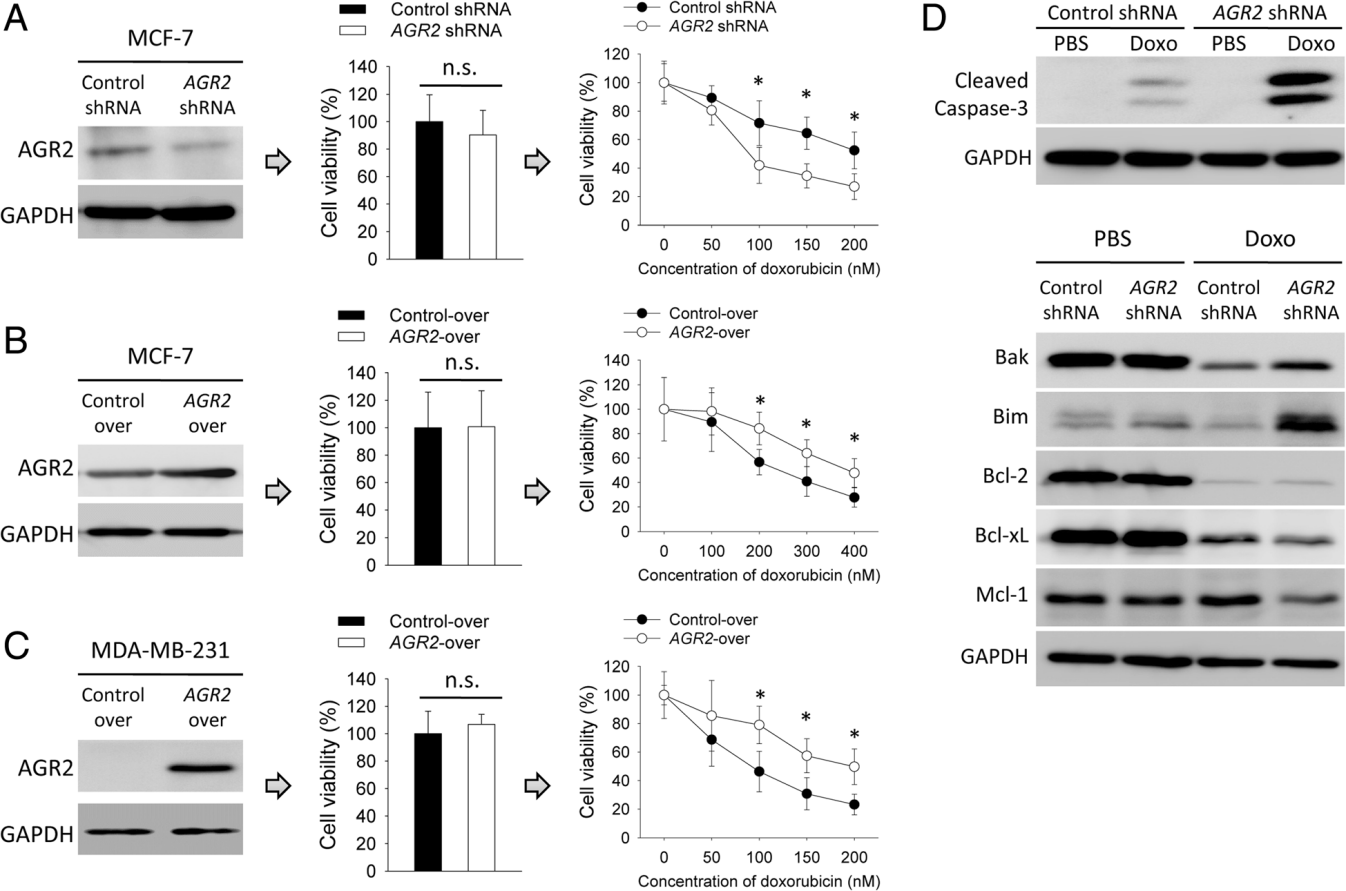
AGR2 mediated doxorubicin-sensitivity of breast cancer cells in vitro. **a** MCF-7 cells were stably transduced with a control shRNA vector or an *AGR2*-shRNA vector. **b** MCF-7 and **c** MDA-MB-231 cells were stably transduced with a control vector or an *AGR2* over-expression vector. **a**-**c** Cells were analyzed for expression of AGR2 protein (left panel). Cells were analyzed for cell viability (middle panel), and viability of control was set as 100%. Cells were treated with doxorubicin for 48 h (right panel), followed by measuring for cell viability (*n* = 6), and viability of untreated cells was set as 100%. **d** MCF-7 cells, stably expressing a control shRNA vector or an *AGR2*-shRNA vector, were treated with doxorubicin (100 nM) for 48 h. Protein levels were detected by western blotting with indicated antibodies. Experiment was repeated three times. Data are shown as mean ± SD, compared using unpaired *t* test.*, *p* < 0.05; n.s., no significance

Next, we examined if AGR2 had an impact on doxorubicin-sensitivity in breast cancer cells, through modulating expression of AGR2 with *AGR2*-shRNA or *AGR2*-over-expression vectors. Knockdown and over-expression of AGR2 increased and decreased sensitivity to doxorubicin in MCF-7 cells, respectively (Fig. 2a and b). Since MDA-MB-231 cells did not expression AGR2 protein, we only constructed over-expression model in this cell line and found that forced expression of AGR2 increased resistance to doxorubicin (Fig. 2c). Impact of AGR2 on apoptosis was further analyzed. Knockdown of AGR2 increased level of cleaved caspase-3 in MCF-7 cells treated by doxorubicin. Knockdown of AGR2 increased level of pro-apoptotic Bak and Bim, but decreased level of anti-apoptotic Mcl-1 (Fig. 2d). These findings indicated that AGR2 mediated drug resistance to doxorubicin in breast cancer cells.

In addition, we also tested the effect of AGR2 in treatment with other chemotherapeutics including paclitaxel, docetaxel and cyclophosphamide in MCF-7 cells. Knockdown of AGR2 increased sensitivity to paclitaxel and docetaxel. However, over-expression of AGR2 did not decrease sensitivity to paclitaxel and docetaxel. In addition, modulating expression of AGR2 had no impact on drug sensitivity to cyclophosphamide (Additional file 1: Figure S1).

### Silence of AGR2 enhanced doxorubicin-sensitivity of MCF-7 cells in vivo

**Fig. 3:**
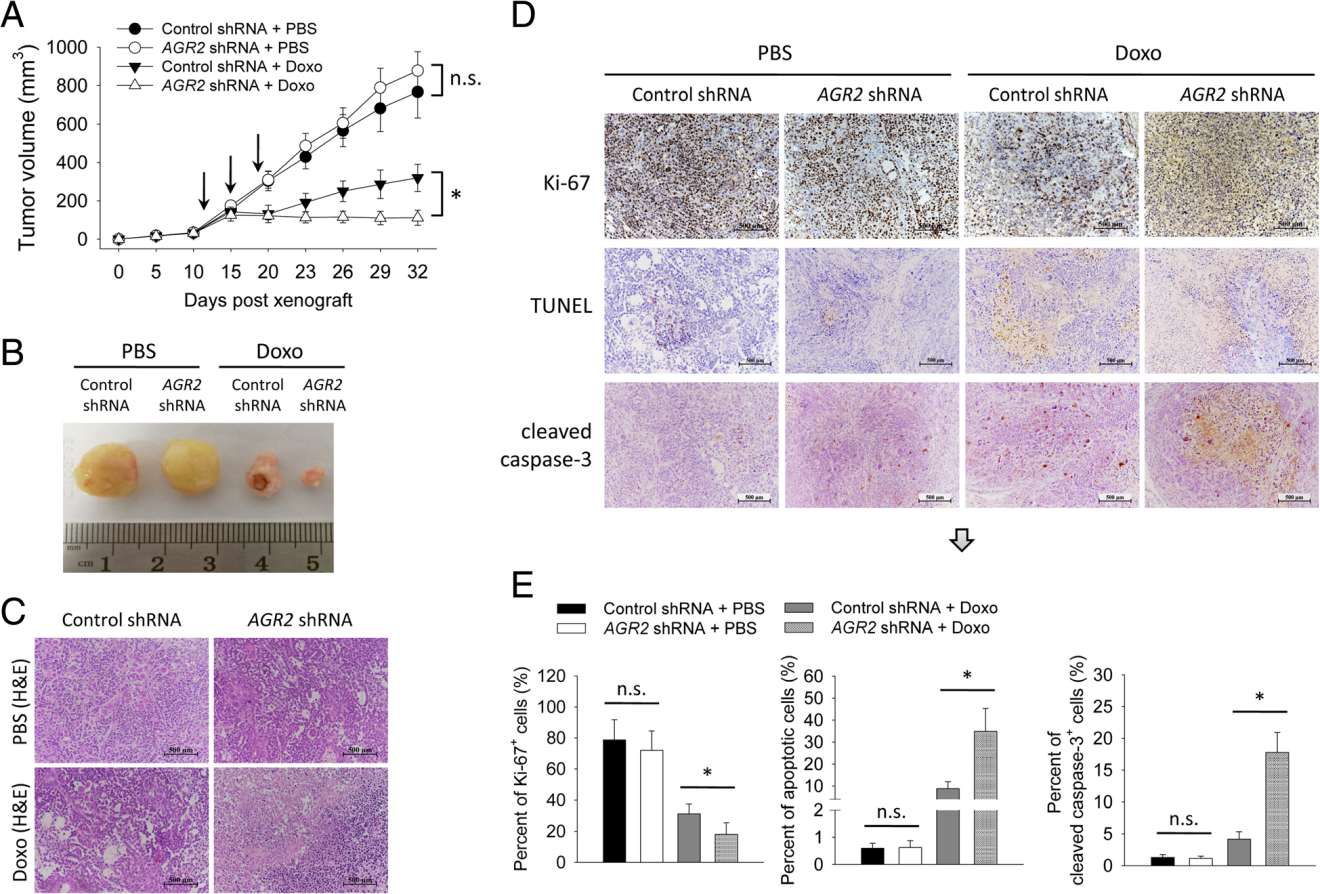
Silence of AGR2 enhanced doxorubicin-sensitivity of MCF-7 cells in vivo. MCF-7 cells, stably transduced with a control shRNA vector or an *AGR2*-shRNA vector, were inoculated subcutaneously into BALB/c Nude mice at a dose of 5 × 10^6^ per mouse (*n* = 4 in each group). Mice were injected intraperitoneally with PBS or doxorubicin (5 mg/kg) once on day 12, 15 and 18 after cell inoculation (indicated by arrows in panel A). **a** Tumor size was measured continuously. **b**-**e** On day 32, tumors were collected from mice followed by morphological (**b**) and histological analyses (**c**). **d**-**e** Immunohistochemistry was performed to detect expression of Ki-67 and cleaved caspase-3 in tumor tissues. TUNEL assay was applied to dectec apoptosis. Scale bar in (**c** and **d**): 500 μm. **e** Percent of Ki-67-positive cells, apoptotic cells and cleaved caspase-3-positive cells were counted. Experiment was repeated twice. Data are shown as mean ± SD, compared using one-way ANOVA test. *, *p* < 0.05; n.s., no significance; Doxo, doxorubicin

To assess the impact of AGR2 on doxorubicin-sensitivity in vivo, we applied a xenograft model in nude mice. *AGR2*-shRNA MCF-7 cells showed a similar growth rate as control-shRNA MCF-7 cells (Fig. 3a and b). After being treated by doxorubicin, *AGR2*-shRNA tumors grew more slowly and were smaller than control-shRNA tumors (Fig. 3a and b). Proliferation and apoptosis in these tumors were further analyzed. *AGR2*-shRNA tumors had less Ki-67-positive cells than control-shRNA tumors after being treated by doxorubicin, whereas *AGR2*-shRNA tumors had more apoptotic cells and higher level of cleaved caspase-3 than control-shRNA tumors after being treated by doxorubicin (Fig. 3c-e). These findings indicated that knockdown of AGR2 increased doxorubicin-sensitivity of MCF-7 cells in vivo in association with increased apoptosis.

### Decreased levels of miR-135b-5p correlated with up-regulation of *AGR2* in breast cancer cells

**Fig. 4:**
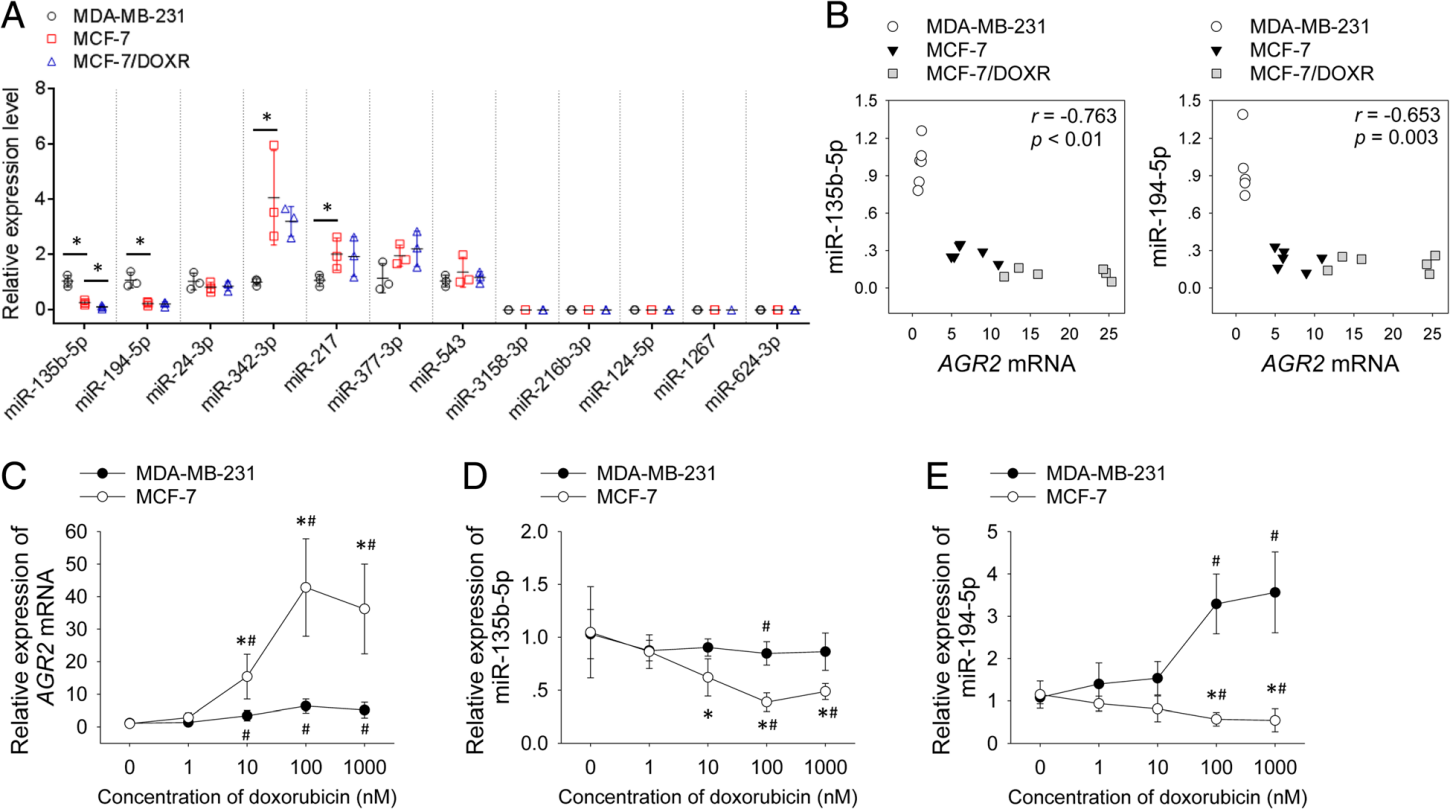
Decreased levels of miR-135b-5p correlated with up-regulation of *AGR2* in breast cancer cells. **a** cDNA was synthesized from total RNA extracted from MDA-MB-231, MCF-7 and MCF-7/DOXR cells. qPCR was applied to measure levels of mature miRNAs with U6 as a normalization gene. **b** Correlation between levels of *AGR2* mRNA and miRNAs were analyzed using *Pearson* correlation test. **c**-**e** MCF-7 and MDA-MB-231 cells were treated with gradient doses of doxorubicin for 48 h. Expression levels of *AGR2* mRNA and miRNAs were measured by qPCR (*n* = 3). Experiment was repeated twice. Expression levels of RNAs represent fold changes. Data are shown as mean ± SD and are compared using unpaired *t* test or one-way ANOVA test. *, *p* < 0.05 compared with MDA-MB-231 cells; #, *p* < 0.05 compared with untreated cells. 0 nM represents untreated cells

Given that miRNAs are potent regulators of AGR2, we predicted several candidate miRNAs targeting *AGR2* mRNA. As we found MCF-7 cells (ER-positive) expressed higher level of AGR2 than MDA-MB-231 (ER-negative) did, we compared levels of candidate miRNAs in these cell lines. ER-expression status is correlated with several miRNAs including miR-342-3p, miR-299-3p, miR-217, miR-190, miR-135b-5p and miR-218 [20]. We found that knockdown of ER-expression increased level of miR-135b-5p but decreased level of miR-342-3p in MCF-7 cells (Additional file 1: Figure S2), so we predicted if those miRNAs would bind 3′-UTR of AGR2 mRNA by searching databases on www.mirbase.org and www.targetscan.org. miR-342-3p, miR-217 and miR-135b-5p were chosen as candidate miRNAs due to their binding potential with 3′-UTR of AGR2 mRNA. We also picked several other high ranked candidate miRNAs including miR-194-5p, miR-543, miR-24-3p, miR-377-3p, miR-3158-3p, miR-216b-3p, miR-124-5p, miR-1267 and miR-624-3p (Additional file 1: Table S1). Expression levels of the latter five miRNAs were undetectable in these cells (Fig. 4a). Comparing with MDA-MB-231 cells, MCF-7 cells expressed lower levels of miR-135b-5p and miR-194-5p. On the contrary, MCF-7 cells had higher levels of miR-342-3p and miR-217 than MDA-MB-231 cells (Fig. 4a). Expression of miR-135b-5p was down-regulated in MCF-7/DOXR cells as compared with MCF-7 cells (Fig. 4a).

Levels of *AGR2* mRNA showed negative correlation with levels of miR-135b-5p and miR-194-5p in these breast cancer cells (Fig. 4b). Since reduced expression of miRNAs lead to up-regulation of target genes [15], we further analyzed correlation between miR-135b-5p or miR-194-5p and AGR2. Short term exposure to doxorubicin induced up-regulation of *AGR2* mRNA both in MCF-7 and MDA-MB-231 cells. MCF-7 cells expressed higher levels of *AGR2* mRNA than MDA-MB-231 cells after doxorubicin treatment (Fig. 4c). Doxorubicin reduced levels of miR-135b-5p both in MCF-7 and MDA-MB-231 cells (Fig. 4d). Doxorubicin reduced levels of miR-194-5p of MCF-7 cells, but increased levels of miR-194-5p of MDA-MB-231 cells (Fig. 4e). These findings indicated that down-regulation of miR-135b-5p correlated with up-regulation of *AGR2* in breast cancer cells.

### miR-135b-5p negatively regulated expression of *AGR2* and increased doxorubicin-sensitivity of MCF-7 cells

**Fig. 5:**
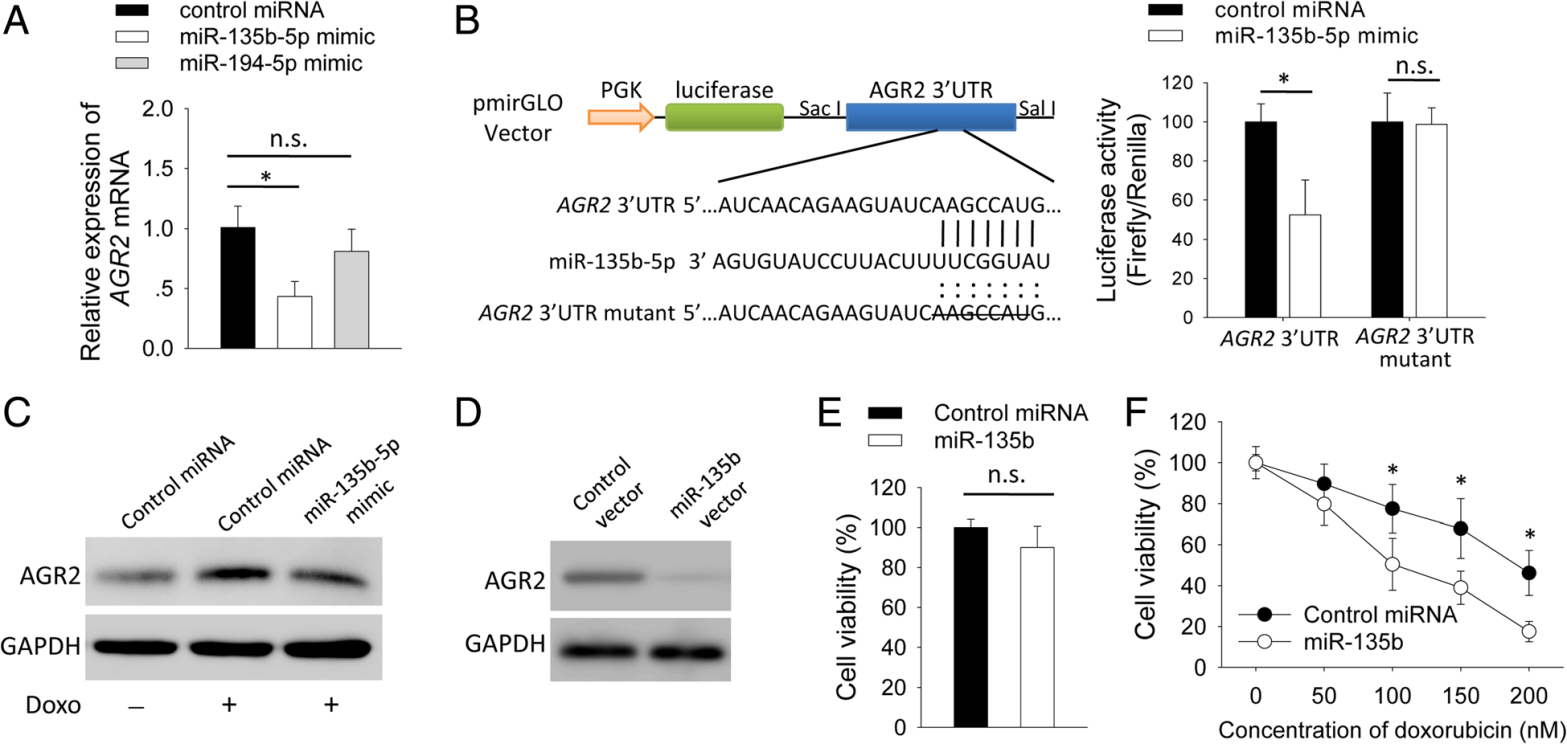
miR-135b-5p negatively regulated expression of *AGR2* and increased doxorubicin-sensitivity of MCF-7 cells in vitro. **a** MCF-7 cells were transfected with mimics of a control miRNA, miR-135b-5p or miR-194-5p. Twenty-four hours after transfection, level of *AGR2* mRNA was measured by qPCR (*n* = 3). **b** *AGR2* 3′-UTR (1097 bp) and a mutant *AGR2* 3′-UTR (1090 bp) fragments were synthesized and cloned to pmirGLO Dual-Luciferase miRNA Target Expression Vector. Luciferase vector and miR-135b-5p mimic were co-transfected into MCF-7 cells. Twenty-four hours after transfection, firefly and renilla luciferase activities of cell lysate were analyzed. Luciferase activity was expressed as ratio of Firefly/Renilla. **c** MCF-7 cells were transfected with mimics of a control miRNA or miR-135b-5p, followed by treatment with doxorubicin (100 nM) for 48 h. AGR2-expression was detected using western blot. **d**-**e** MCF-7 cells were stably transduced with lentivirual vector expressing double strand precursors of a control miRNA or miR-135b. **d** AGR2-expression was detected using western blot. **e** Cell viability was measured by CCK-8 assay (*n* = 6). **f** Cells were treated with doxorubicin for 48 h followed by measuring for cell viability (*n* = 6). Experiment was repeated three times. Expression levels of mRNA represent fold changes. Data are shown as mean ± SD and are compared using unpaired *t* test or one-way ANOVA test. *, *p* < 0.05; n.s., no significance

Through transfecting MCF-7 cells with miRNAs mimics, we found that miR-135b-5p mimic reduced levels of *AGR2* mRNA, whereas miR-194-5p had no impact on levels of *AGR2* mRNA (Fig. 5a). Next, we analyzed if miR-135b-5p targeted 3′-UTR of *AGR2* mRNA. As MCF-7 cells expressed a relative low level of background miR-135b-5p, MCF-7 cells were used as transfection host of a luciferase reporter vector expressing 3′-UTR of *AGR2* mRNA. Co-transfection with miR-135b-5p mimic suppressed luciferase activity, compared with a negative control miRNA. We also constructed a mutant *AGR2* 3′-UTR luciferase reporter vector by deleting the binding site of miR-135b-5p, and found that miR-135b-5p mimic failed to suppress luciferase activity of cells transfected with the mutant vector (Fig. 5b). Doxorubicin-treatment reduced levels of miR-135b-5p of MCF-7 cells (Fig. 4d). To test if doxorubicin induced down-regulation of miR-135b-5p is a probable reason for over-expression of AGR2, cells were transfected with miR-135b-5p mimic before treatment with doxorubicin. Doxorubicin induced up-regulation of AGR2 level was reduced by miR-135b-5p mimic (Fig. 5c).

**Fig. 6:**
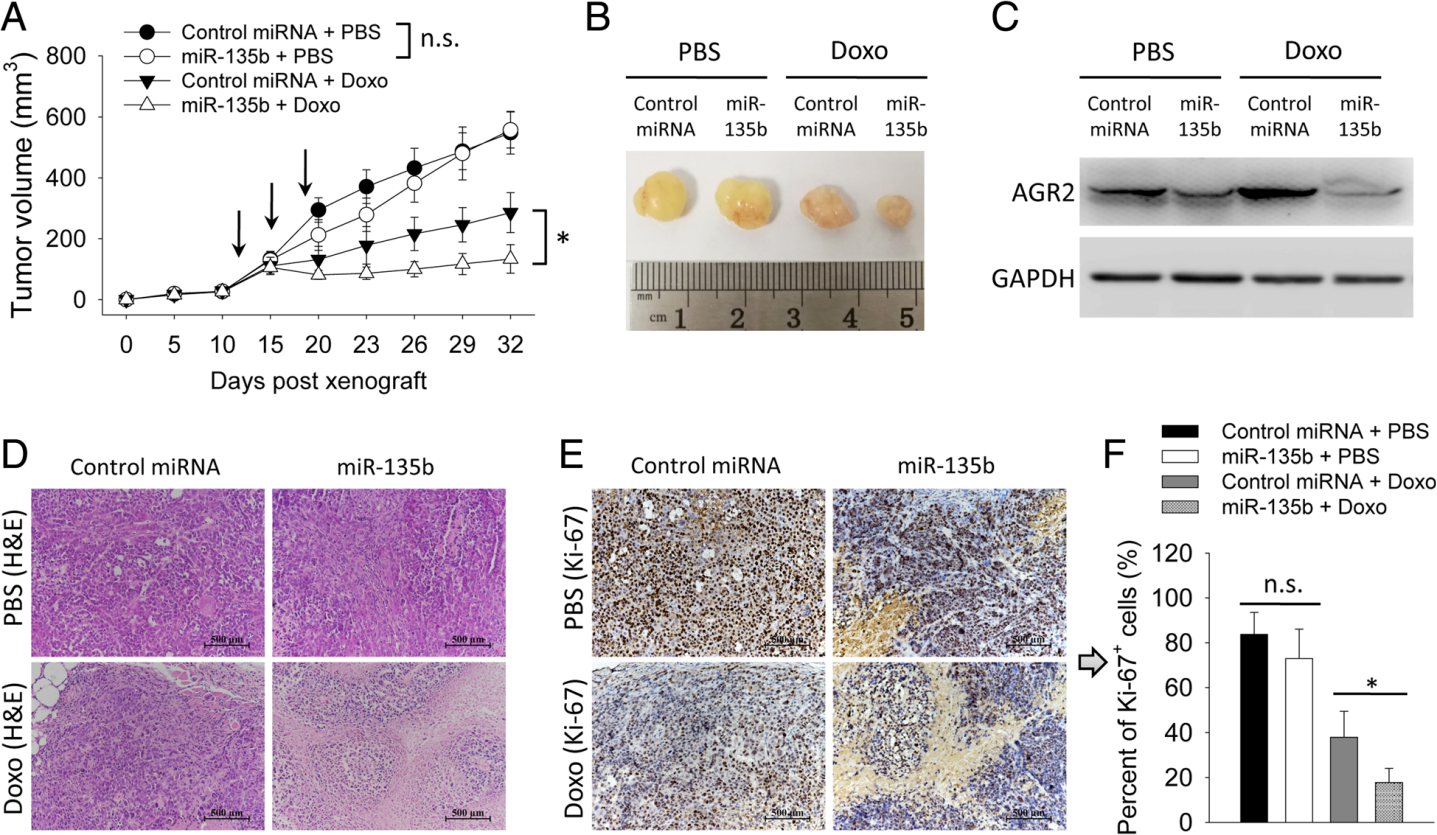
miR-135b-5p increased doxorubicin-sensitivity of MCF-7 cells in vivo. MCF-7 cells, stably transduced with lentivirual vector expressing double strand precursors of a control miRNA or miR-135b, were inoculated subcutaneously into BALB/c Nude mice at a dose of 5 × 10^6^ per mouse (*n* = 4 in each group). Mice were injected intraperitoneally with PBS or doxorubicin (5 mg/kg) once on day 12, 15 and 18 after cell inoculation (indicated by arrows in panel A). **a** Tumor size was measured continuously. **b**-**f** On day 32, tumors were collected from mice. **c** Proteins extracted from tumors were analyzed by western blotting with indicated antibodies. **d** Histological analysis was performed using H&E staining. **e** Immunohistochemistry was performed to detect Ki-67-expression in tumor tissues. Scale bar in (**d** and **e**): 500 μm. **f** Percent of Ki-67-positive cells was counted. Experiment was repeated twice. Data are shown as mean ± SD, compared using one-way ANOVA test. *, *p* < 0.05; n.s., no significance

Furthermore, we up-regulated expression of miR-135b-5p in MCF-7 cells by transducing cells with a lentivirus vector expressing double strand miR-135b precursor. Transduction of miR-135b vecter reduced expression of AGR2 (Fig. 5d). Transduction of miR-135b vecter had no impact on viability of cells in the absence of doxorubicin (Fig. 5e). Forced expression of miR-135b-5p in MCF-7 cells increased doxorubicin-sensitivity as compared with negative control (Fig. 5f). Next, we tested the impact of miR-135b-5p on doxorubicin-sensitivity in the murine xenograft model. The miR-135b vecter-transduced MCF-7 cells showed a similar growth rate as control (Fig. 6a and b). Transduction of miR-135b vecter reduced level of AGR2 in the tumors (Fig. 6c). After being treated by doxorubicin, miR-135b tumors grew more slowly (Fig. 6a and b) and had less Ki-67-positive cells (Fig. 6d-f) than control. These findings confirmed that miR-135b-5p negatively regulated expression of *AGR2* through targeting 3′-UTR and increased doxorubicin-sensitivity of MCF-7 cells.

### Levels of miR-135b-5p negatively correlated with levels of AGR2 in clinical breast cancer samples

**Fig. 7:**
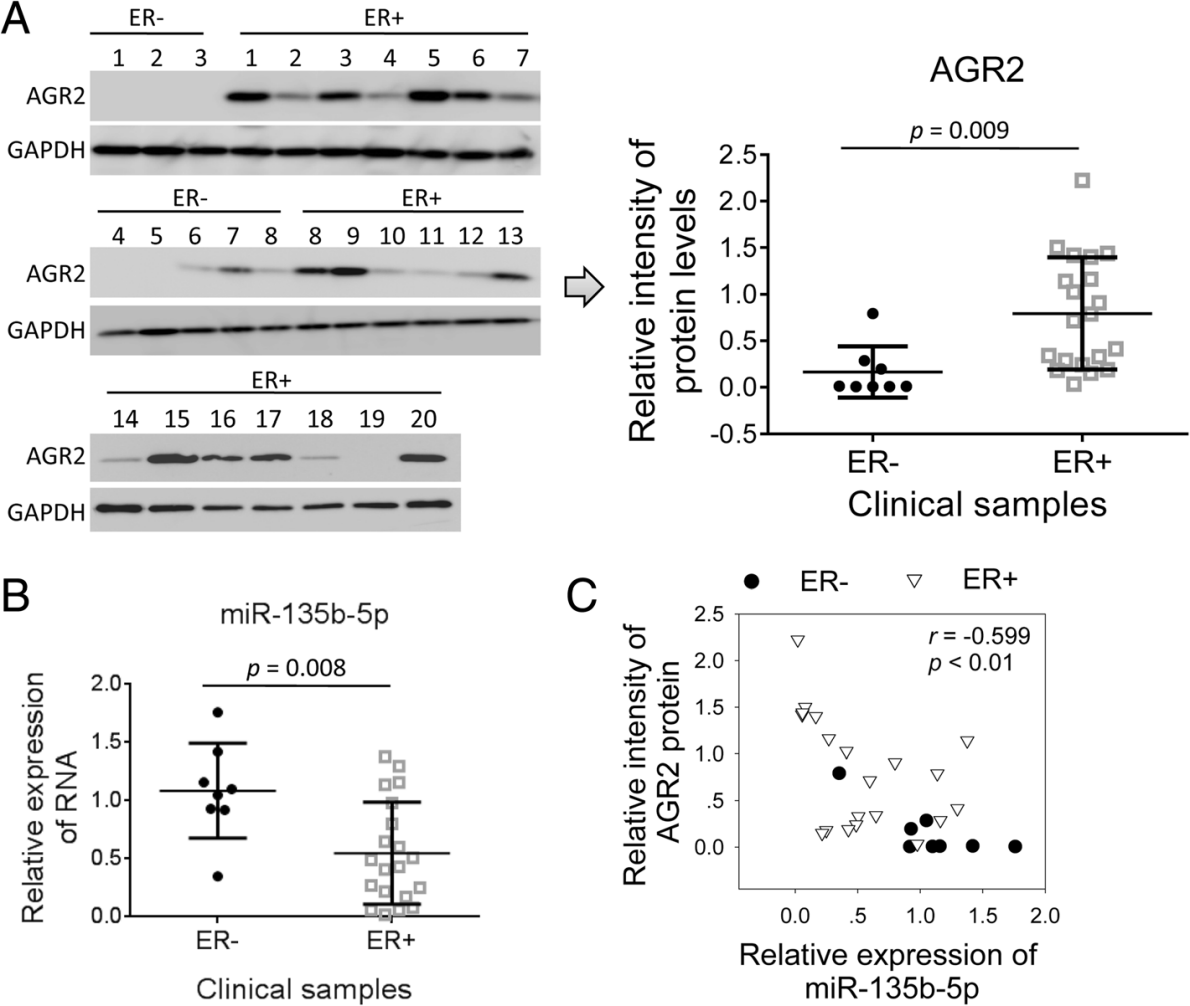
Levels of miR-135b-5p negatively correlated with levels of AGR2 in clinical breast cancer tissues. Twenty-eight breast cancer tissue samples were collected from patients. Total RNA and protein were extracted from tissues. **a** AGR2-expression was detected using western blot. Intensities of AGR2-bands were analyzed with ImageJ software, and were normalized to GAPDH. **b** qPCR was applied to measure levels of miR-135b-5p with U6 as a normalization gene. Expression levels of RNA represent fold changes. Data are shown as mean ± SD and are compared using unpaired *t* test. **c** Correlation between levels of AGR2 protein and miR-135b-5p was analyzed using *Pearson* correlation test

Clinical breast cancer samples were collected and subjected to analysis for expression of AGR2 protein and miR-135b-5p. ER-positive tumors (*n* = 20) expressed higher levels of AGR2 than ER-negative ones (*n* = 8). On the contrary, ER-positive tumors showed lower levels of miR-135b-5p comparing with ER-negative ones (Fig. 7a and b). Although expression of AGR2 was reported to be regulated by ER, we observed that expression of AGR2 are at low levels or undetectable in some ER-positive samples. In addition, ER-negative sample #6, #7 and #8 expressed low or middle levels of AGR2 (Fig. 7a). Interestingly, we observed a negative correlation between levels of AGR2 and miR-135b-5p in these samples (Fig. 7c). These findings indicated that level of miR-135b-5p might be a predictive marker for AGR2-expression in breast cancer.

## Discussion

AGR2 is a protein disulphide isomerase regulating protein folding in endoplasmic reticulum which is important for survival of tumor cells, as many tumor cells produce abundant proteins which would cause proteotoxic stress to cells in case of insufficient or inappropriate protein-folding [7, 21]. Importantly, AGR2 was implied to mediate drug resistance in breast cancer [7, 10, 11]. Expression level of AGR2 correlated with doxorubicin-sensitivity of breast cancer cells in our study. Up-regulation and down-regulation of AGR2 decreased and increased doxorubicin-sensitivity, respectively (Fig. 2), and this effect is associated with apoptotic process. Knockdown of AGR2 increased apoptosis and reduced proliferation of the xenograft tumors (Fig. 3). AGR2 was shown to induce expression of cyclin D1 in breast cancer cells [22]. Cyclin D1 is a critical driver of tumorigenesis of breast cancer [23, 24]. On the other hand, dysregulation of cyclin D1 is a marker of senescence, which counteracts tumor genesis [25, 26]. However, exposure to doxorubicin had no impact on senescence or expression of cyclin D1 in our study (Additional file 1: Figure S3). Li and colleague had shown that AGR2 stabilized hypoxia inducible factor-1α enhancing hypoxia-induced doxorubicin resistance in breast cancer cells [11]. Over-expression of AGR2 alone seems insufficient for tumorigenesis of breast cancer [27], but rather can contribute to drug resistance. Consistently, our results showed that knockdown or over-expression of AGR2 in MCF-7 and MDA-MB-231 cells had no significant impact on cell viability in the absence of doxorubicin. AGR2 mediated drug resistance seems more related with topoisomerase II inhibitor. Knockdown of AGR2 increased sensitivity to paclitaxel and docetaxel. However, over-expression of AGR2 had no impact on sensitivity to these drugs in MCF-7 cells (Additional file 1: Figure S1). AGR2 also mediates resistance to hormone therapy in breast cancer [9]. All these suggest that manipulating expression of AGR2 might counteract drug-resistance in breast cancer, especially in those who with high level of AGR2.

Others reported that level of AGR2 positively correlated with expression of ER in breast cancer. ER binds directly to AGR2 promoter to activate transcription of AGR2 in both cell lines and tumor samples of breast cancers [12, 13]. AKT signaling pathway was also reported to induce AGR2 expression [28]. Our data on clinical breast cancer samples also showed that ER-positive tumors expressed higher levels of AGR2 than ER-negative ones (Fig. 7). MCF-7 and MDA-MB-231 cells are ER-positive and ER-negative cells, respectively. Thus, expression of ARG2 in these two cell lines might have correlation with expression of ER (Fig. 1). Some clinical data showed that over-expression of AGR2 can also be found in ER-negative breast cancers [10], which indicates that other mechanisms might also participate in regulating AGR2-expression. Interestingly, we found expression of AGR2 could be further regulated by miR-135b-5p.

miRNAs play important roles in multiple cellular processes such as proliferation and differentiation [29, 30]. Prognostic miRNAs, such as miR-342 and miR-30e, were reported in breast cancer and some miRNAs were proposed as predictive markers of drug resistance [16, 31, 32]. However, miRNAs-mediated regulation of AGR2 is still unclear in breast cancer. Since AGR2 mediates doxorubicin-resistance, we further focused on miRNAs-mediated regulation of AGR2. Among these candidate miRNAs, down-regulation of miR-135b-5p was in parallel with up-regulation of AGR2 in breast cancer cells. Doxorubicin-treatment induced up-regulation of AGR2 in association with down-regulation of miR-135b-5p (Fig. 4). miR-135b-5p negatively regulated expression of *AGR2* through targeting 3′-UTR confirmed that AGR2 is a target gene of miR-135b-5p in breast cancer cells (Fig. 5). As expected, miR-135b-5p reduced expression of AGR2 and increased doxorubicin-sensitivity of MCF-7 cells both in vitro and in vivo (Figs. 5 and 6). Doxorubicin treatment induced down-regulation of miR-135b-5p is a probable reason for over-expression of AGR2 in breast cancer (Figs. 4 and 5), which is indicative for a treatment related drug resistance. Intriguingly, we also observed a negative correlation between levels of AGR2 and miR-135b-5p in clinical breast cancer samples (Fig. 7). Level of miR-194-5p also negatively correlated with level of AGR2 in breast cancer cell lines. Interestingly, doxorubicin treatment increased level of miR-194-5p in MDA-MB-231 cells (Fig. 4). Expression of miR-194-5p is regulated by some upstream regulators such as GATA2 and non-coding RNAs [33, 34]. The difference on levels of miR-194-5p in MCF-7 and MDA-MB-231 might have some relations with ER or those upstream regulators of miR-194-5p. In addition, transfection with miR-194-5p did not down-regulate expression of AGR2 (Fig. 5). miR-194-5p might not be a direct regulator of AGR2 in breast cancers. Thus, in addition to ER-dependant mechanism, we identified that miR-135b-5p is another regulator of AGR2 in breast cancer.

Others reported that increased levels of miR-342-3p and miR-217 positively correlated with expression of ER in breast cancer [20]. Consistently, we showed MCF-7 had higher levels of miR-342-3p and miR-217 than MDA-MB-231. Their relations with AGR2 were reported by others recently. miR-342-3p targets AGR2 and down-regulation of miR-342-3p is associated with over-expression of AGR2 in non-small cell lung cancer [35]. Down-regulation of miR-217 was associated with over-expression of AGR2 in chronic myelogenous leukemia cells [36]. Doxorubicin-treatment had no impact on level of miR-342-3p or miR-217 in breast cancer cells (data not shown). Since levels of miR-342-3p and miR-217 positively correlated with level of AGR2 in breast cancer cells, we deduce that miR-135b-5p is a more dominant regulator of AGR2-expression in breast cancer, comparing with miR-342-3p and miR-217. miR-1291 was shown to regulate expression of AGR2 in pancreatic cancer cell [37]. The findings suggest that miRNAs mediated regulation on AGR2 differs in different type of cancers.

## Conclusion

In conclusion, AGR2 is a target of miR-135b-5p. Decreased levels of miR-135b-5p correlated with over-expression of AGR2 in breast cancer cells during doxorubicin treatment. Up-regulation of miR-135b-5p suppressed expression of AGR2 increasing doxorubicin-sensitivity of breast cancer cells. Our results highlight the potential of miR-135b-5p as a target for treating AGR2-expressing breast cancer with doxorubicin-resistance.

## Additional file

Additional file 1: **Table S1.** Candidate miRNAs with AGR2-targeting potential. **Figure S1.** Knockdown of AGR2 increased sensitivity to paclitaxel and docetaxel in MCF-7 cells. **Figure S2.** Knockdown of estrogen receptor 1 (ESR1) increased level of miR-135b-5p but decreased level of miR-342-3p in MCF-7 cells. **Figure S3.** Long term exposure to doxorubicin had no impact on senescence. (DOCX 1106 kb)

## Abbreviations

AGR2: Anterior gradient 2
DOXR: Doxorubicin resistant
ER: Estrogen receptor
miRNAs: microRNAs
PDIs: Protein disulphide isomerases
shRNA: Short hairpin RNA
UTR: Untranslated region
