# Supplementary material for: miR-135b-5p enhances doxorubicin-sensitivity of breast cancer cells through targeting anterior gradient 2
Source: J Exp Clin Cancer Res. 2019 Jan 21;38:26. doi: 10.1186/s13046-019-1024-3 (PMC6341729; doi:10.1186/s13046-019-1024-3)
Supplement: Supplementary file 1 — Table S1. Candidate miRNAs with AGR2-targeting potential. Figure S1. Knockdown of AGR2 increased sensitivity to paclitaxel and docetaxel in MCF-7 cells. Figure S2. Knockdown of estrogen receptor 1 (ESR1) increased level of miR-135b-5p but decreased level of miR-342-3p in MCF-7 cells. Figure S3. Long term exposure to doxorubicin had no impact on senescence. (DOCX 1106 kb) [file 13046_2019_1024_MOESM1_ESM.docx]

**Supplemental data**

**Table S1. Candidate miRNAs with AGR2-targeting potential**

| No. | miRNA name | Sequence (5’->3’) | Predicted binding position of AGR2 3'-UTR | Site type | Context++ score percentile |
| --- | --- | --- | --- | --- | --- |
| 1 | miR-342-3p | UCUCACACAGAAAUCGCACCCGU | 60-67 | 8mer | 99 |
| 2 | miR-217 | UACUGCAUCAGGAACUGAUUGGA | 1040-1046 | 7mer-A1 | 86 |
| 3 | miR-135b-5p | UAUGGCUUUUCAUUCCUAUGUGA | 456-462 | 7mer-m8 | 88 |
| 4 | miR-194-5p | UGUAACAGCAACUCCAUGUGGA | 120-126 | 7mer-A1 | 94 |
| 5 | miR-543 | AAACAUUCGCGGUGCACUUCUU | 118-124 | 7mer-A1 | 90 |
| 6 | miR-24-3p | UGGCUCAGUUCAGCAGGAACAG | 210-216 | 7mer-A1 | 93 |
| 7 | miR-377-3p | AUCACACAAAGGCAACUUUUGU | 179-186 | 8mer | 99 |
| 8 | miR-3158-3p | AAGGGCUUCCUCUCUGCAGGAC | 16-22 | 7mer-m8 | 93 |
| 9 | miR-216b-3p | ACACACUUACCCGUAGAGAUUCUA | 58-65 | 8mer | 99 |
| 10 | miR-124-5p | CGUGUUCACAGCGGACCUUGAU | 92-99 | 8mer | 98 |
| 11 | miR-1267 | CCUGUUGAAGUGUAAUCCCCA | 442-449 | 8mer | 99 |
| 12 | miR-624-3p | CACAAGGUAUUGGUAUUACCU | 719-726 | 8mer | 98 |


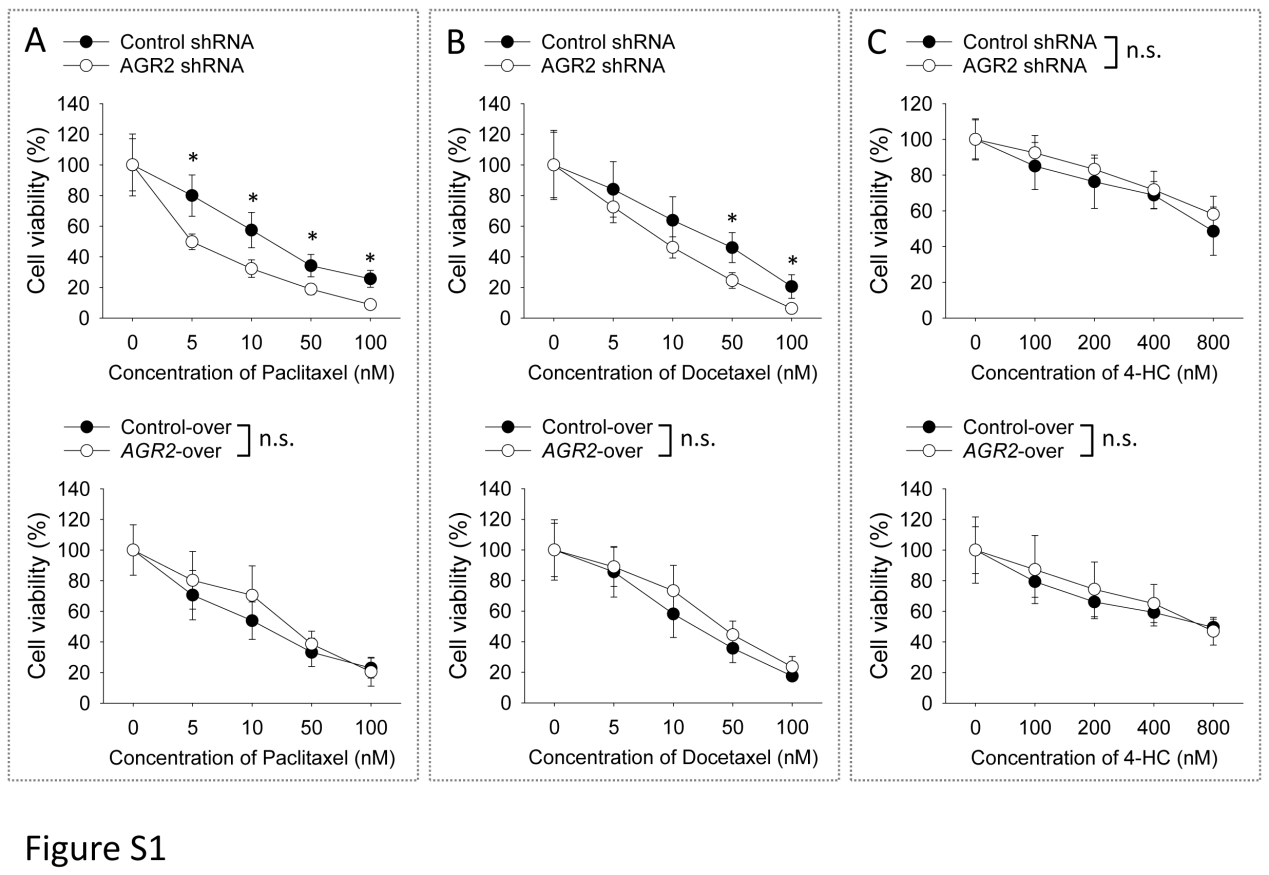


**Figure S1.** **Knockdown of AGR2 increased sensitivity to paclitaxel and docetaxel in MCF-7 cells.** MCF-7 cells, stably expressing a control vector, an *AGR2*-shRNA vector or an *AGR2* over-expression vector, were treated with different doses paclitaxel (**A**), docetaxel (**B**) and 4-hydroperoxy cyclophosphamide (4-HC) (**C**) for 24 hours. Cell viability was measured by CCK-8 assay (n=6). Experiment was repeated twice. Data are shown as mean ± SD, compared using unpaired *t* test.*, *p* < 0.05; n.s., no significance.

**
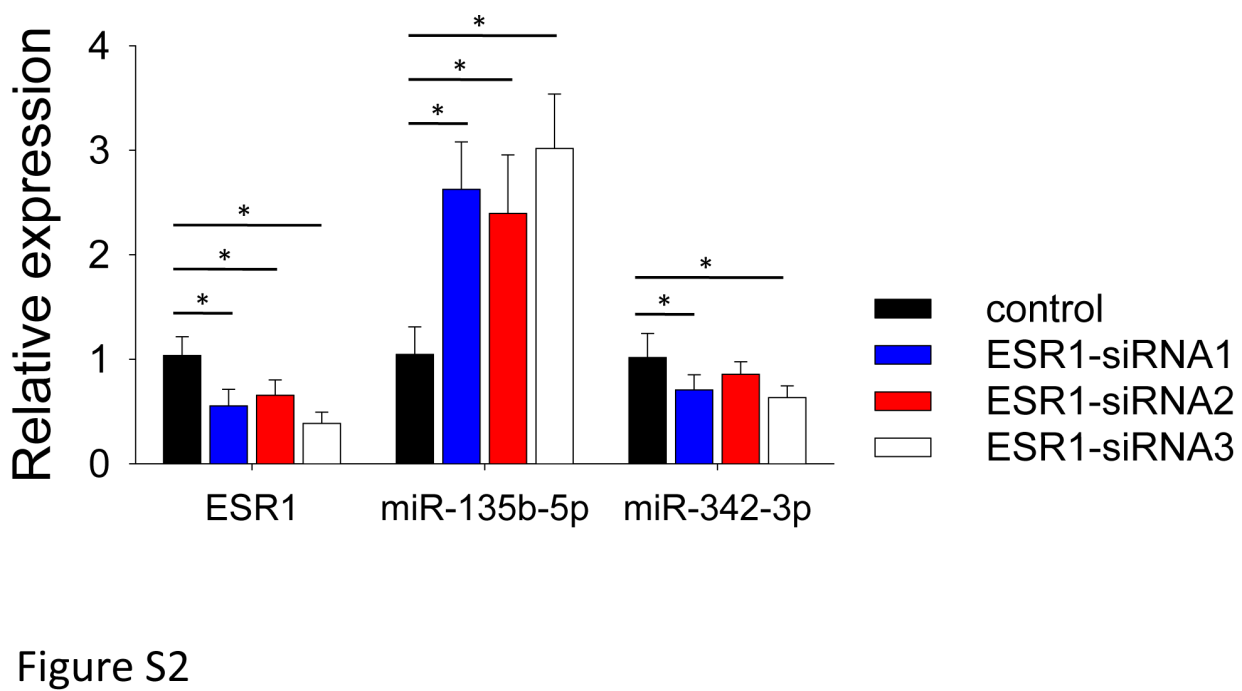
**

**Figure S2.** **Knockdown of estrogen receptor 1 (****ESR1) increased level of** **miR-135b-5p but decreased level of** **miR-342-3p in MCF-7 cells.** MCF-7 cells were transfected with *ESR1*-targeting siRNAs or a negative control RNA. Total RNAs were isolated 24 h after transfection. Levels of miR-135b-5p and miR-342-3p were analyzed using qPCR with U6 as a normalization gene (n=3). Expression levels represent fold changes. Data are shown as mean ± SD, compared using one-way ANOVA test. *, *p* < 0.05.

**
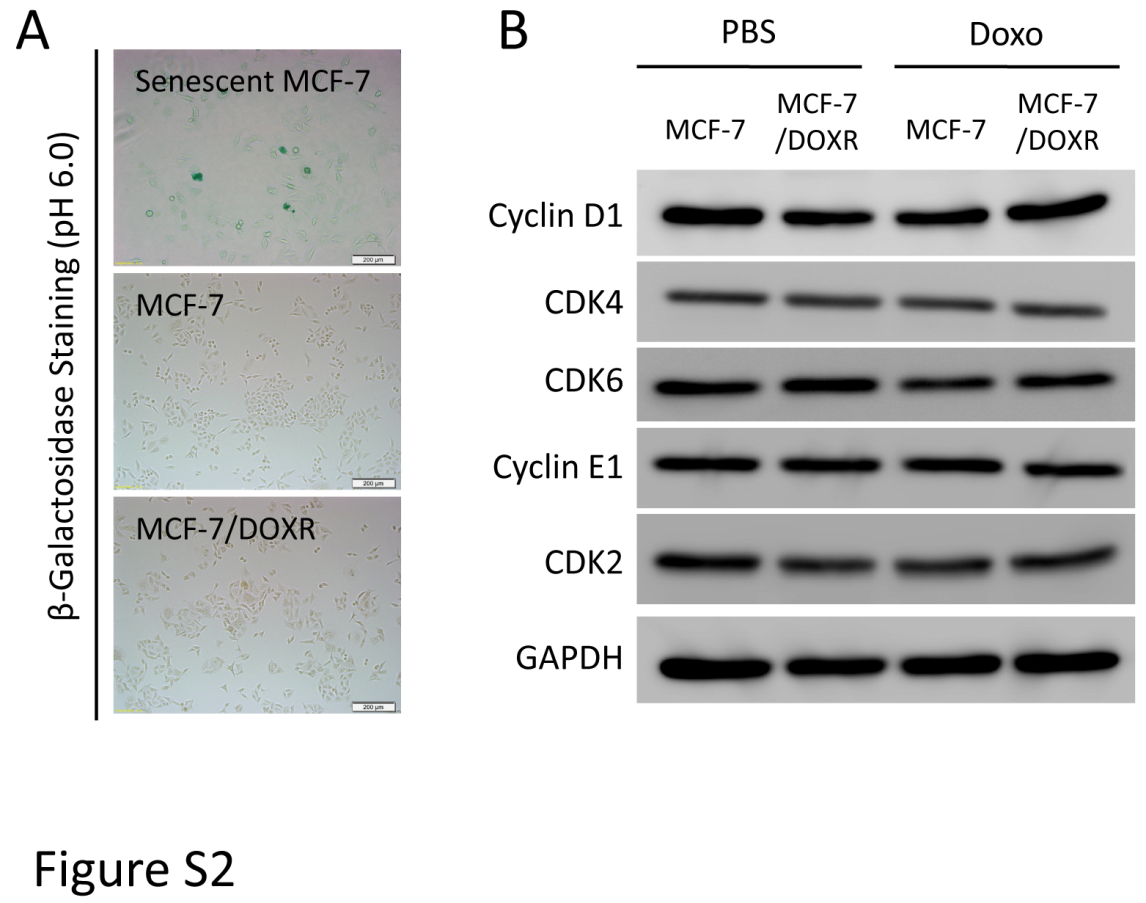
**

**Figure S3. Long term** **exposure to doxorubicin had no impact on senescence.** Doxorubicin-resistant MCF-7 cells (MCF-7/DOXR) were selected through sequential exposure to increasing doses of doxorubicin (0.1, 0.5, 1.0, 2.0 and 5.0 μM). (**A**) Cells were selected for two months followed by staining with a β-Galactosidase Staining Kit. Senescent MCF-7 cells and untreated MCF-7 cells were used as control. Scale bar: 200 μm. (**B**) Untreated MCF-7 and MCF-7/DOXR cells were treated with PBS or doxorubicin (100 nM) for 48 hours, followed by western blotting with indicated antibodies. Experiment was repeated three times.
